# The roles of CC chemokines in response to radiation

**DOI:** 10.1186/s13014-022-02038-x

**Published:** 2022-04-01

**Authors:** Lei Wang, Jizong Jiang, Yuan Chen, Qingzhu Jia, Qian Chu

**Affiliations:** 1 Department of Oncology, Tongji Hospital, Tongji Medical College, Huazhong University of Science and Technology, 1095 Jiefang Avenue, Wuhan, 430030 People’s Republic of China; 2 Chongqing Key Laboratory of Immunotherapy, Chongqing, People’s Republic of China; 3 Department of Oncology, Xinqiao Hospital, Third Military Medical University, Chongqing, People’s Republic of China

**Keywords:** Radiotherapy, Chemokine, CC chemokine, Tumour microenvironment, Radiation-induced injury, Protumour effect, Antitumour effect

## Abstract

Radiotherapy is an effective regimen for cancer treatment alone or combined with chemotherapy or immunotherapy. The direct effect of radiotherapy involves radiation-induced DNA damage, and most studies have focused on this area to improve the efficacy of radiotherapy. Recently, the immunomodulatory effect of radiation on the tumour microenvironment has attracted much interest. Dying tumour cells can release multiple immune-related molecules, including tumour-associated antigens, chemokines, and inflammatory mediators. Then, immune cells are attracted to the irradiated site, exerting immunostimulatory or immunosuppressive effects. CC chemokines play pivotal roles in the trafficking process. The CC chemokine family includes 28 members that attract different immune subsets. Upon irradiation, tumour cells or immune cells can release different CC chemokines. Here, we mainly discuss the importance of CCL2, CCL3, CCL5, CCL8, CCL11, CCL20 and CCL22 in radiotherapy. In irradiated normal tissues, released chemokines induce epithelial to mesenchymal transition, thus promoting tissue injury. In the tumour microenvironment, released chemokines recruit cancer-associated cells, such as tumour-infiltrating lymphocytes, myeloid-derived suppressor cells and tumour-associated macrophages, to the tumour niche. Thus, CC chemokines have protumour and antitumour properties. Based on the complex roles of CC chemokines in the response to radiation, it would be promising to target specific chemokines to alleviate radiation-induced injury or promote tumour control.

## Background

RT is administered as a curative or palliative therapy in more than 50% of cancer patients [1]. The most frequent and lethal lesions that occur during radiation include single-strand breaks (SSBs), double-strand breaks (DSBs), DNA-DNA or DNA–protein cross-links, base release and other chemical modifications, and multiple damaged sites in DNA [2, 3]. Most research on improving the efficacy of RT has focused on tumour cells [4]. However, tumour lesions are not purely composed of cancer cells, and multiple components are present with cancer cells. These components include (1) immune cells; (2) fibroblasts and epithelial cells; (3) extracellular matrix proteins; (4) blood and lymphatic vessels; and (5) metabolites, chemokines, and cytokines [5]. RT can influence these components in different manners, thus remodelling the tumour microenvironment. In tumour tissues, radiation could induce local release of inflammatory cytokines, temporarily eradicate local radiation-sensitive immune cell lineages, and promote immune cell trafficking and immune cell activation [6]. Immune cell subtypes, including dendritic cells (DCs) or cytotoxic lymphocytes, promote antitumour immunity. However, suppressive cells could also be attracted by radiation. In these contexts, chemokines play fundamental roles.

**Fig. 1:**
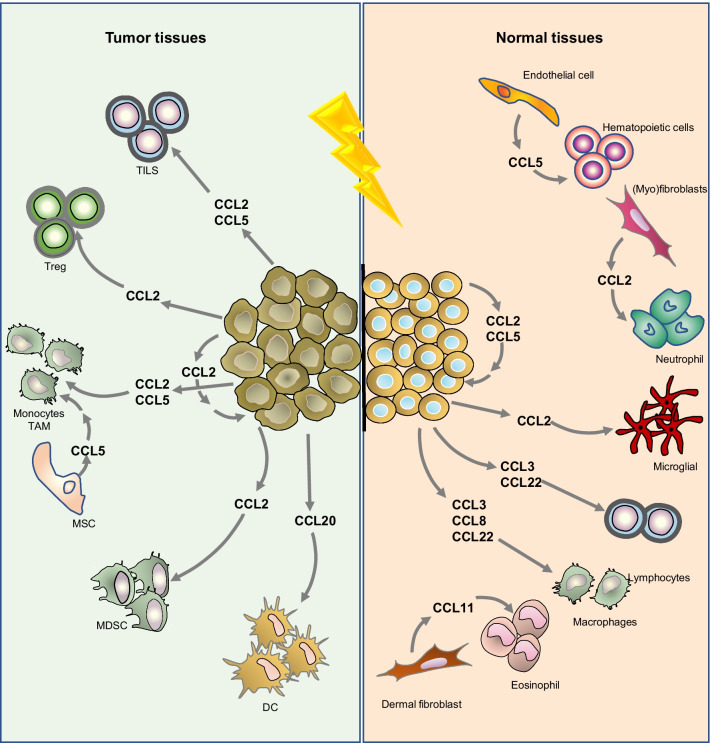
CC chemokines in radiation responses. The figure shows the chemokines released by tumour cells or adjacent normal cells. In tumour tissues (left part, green), the released chemokines could act on tumour cells or attract different immune subsets, which could promote tumour control or metastasis. In normal tissues (right part, red), radiation promotes the production of chemokines in epithelial cells, endothelial cells or fibroblasts. The released chemokines could trigger EMT in primary cells. Multiple immune cell subsets could be recruited, including macrophages, lymphocytes, eosinophils, neutrophils and microglia. These outcomes ultimately lead to radiation-induced injury. Abbreviations: MSC, MSCs; MDSCs, myeloid-derived suppressor cells; TAMs, tumour-associated macrophages; TILs, tumour-infiltrating lymphocytes; DC, dendritic cells; Treg, regulatory T lymphocytes

Chemokines include different subfamilies according to the domain found at the N-terminus. CC chemokines are one of these families and have an N-terminal CC domain [7, 8]. CC chemokines are represented using 28 symbols. The chemokines C–C motif chemokine ligand (CCL)9 and CCL10 are the same chemokine; thus, there are a total of 27 CC chemokines [7, 8]. These chemokines form a complex network that influences the distribution of immune cells in the tumour microenvironment [9]. Although the role of CC chemokines in cancer has been discussed elsewhere, the relationship between radiation and these chemokines has not been described. The aim of this review was to collect information about the involvement of each CC chemokine in radiation (Fig. 1).

## CCL2

CCL2, also known as monocyte chemoattractant protein (MCP)-1, can bind to multiple receptors, including CC chemokine receptor (CCR)1 [10], CCR2 [11], CCR4 [12] and CCR5 [13]. CCL2 is also an antagonist of CCR3 [14]. The main function of CCL2 involves the recruitment of monocytes to inflammatory sites [15–17]. In tumours, CCL2 can be secreted by multiple cell types, including tumour cells [18–21], myeloid-derived suppressor cells (MDSCs) [22], mesenchymal stem cells (MSCs) [23], tumour-associated macrophages (TAMs) [19, 24–26], tumour-associated neutrophils (TANs) [27], and cancer-associated fibroblasts (CAFs) [28–30]. It has been reported that RT can induce the expression of CCL2 [31, 32]. In this section, we will discuss the significance of CCL2 in radiation-related events.

### CCL2 is involved in the radiation-induced immune response

#### Promotion of macrophage infiltration

As mentioned previously, one of the most important functions of CCL2 is the recruitment of monocytes. It has been reported that radiation can promote macrophage infiltration in lung cancer. This infiltration may promote tumour progression through the induction of EMT [33] or triggering an immunosuppressive microenvironment [34]. IL-6 was shown to play a positive role in radiation-induced macrophage migration. When IL-6 signalling was blocked, radiation-induced CCL2 upregulation was inhibited. Neutralizing CCL2 significantly reduced the number of migrated macrophages, indicating that CCL2 was downstream of IL-6 signalling and mediated radiation-induced macrophage infiltration [35]. In the radioresistant pancreatic ductal adenocarcinoma (PDAC) model, tumour-derived CCL2 was significantly upregulated, resulting in increased infiltration of inflammatory monocytes and macrophages but not T cells. When CCL2 was blocked by a specific antibody, the recruitment of inflammatory monocytes was impaired, and tumour growth was delayed [36].

#### Recruitment of MDSCs

Radiation can induce the recruitment of MDSCs. These CCR2-expressing MDSCs contribute to radioresistance. Using CCR2-knockout mice or CCR2-depleting antibodies can reverse the inhibitory effect of MDSCs. The recruitment of MDSCs was further shown to be dependent on activation of the STING pathway. The activated STING pathway promoted the production of type I IFN, which induced CCL2, CCL7, and CCL12 expression, mobilizing monocytes to tumours [37].

#### Recruitment of T cells

In addition to the effect on monocytes, CCL2 is involved in the recruitment of T cells. In a sarcoma mouse model, 20 Gy radiation upregulated CCL2 expression. This outcome was associated with the activation and increased infiltration of Th1/Tc1 T cells in the tumour microenvironment [38]. In a murine model of head and neck squamous cell carcinoma, mice received 7.5 Gy radiation. Irradiation upregulated CCL2 production, promoting the infiltration of tumour necrosis factor-alpha (TNFα)-producing monocytes and CCR2^+^ regulatory T cells (Tregs). In this context, monocyte-derived TNFα activated Tregs, inhibiting the effect of RT [39].

The contradictory effects of CCL2 on antitumour immunity may be dependent on the experimental context, especially radiation dose. A higher dose may stimulate a stronger immune response. In most cases, CCL2 attracts immunosuppressive monocytes, which contribute to tumour progression. This finding suggests that inhibiting CCL2 with radiation may promote the efficacy of RT.

### CCL2 mediates radioresistance

In a nasopharyngeal carcinoma (NPC) model, CCL2 correlated with radioresistance and metastasis. CCL2 expression was significantly elevated in HONE1-IR cells and recurrent NPC tumours. CCL2 could promote adaptive radioresistance, metastasis, and EMT in NPC cells. Inhibiting CCL2 enhanced the sensitivity of NPC cells to radiation. In clinical cohorts, high CCL2 expression could predict poorer distant metastasis-free survival [40].

### CCL2 and radiation-induced toxicity in normal tissue

Although the primary tumour is the direct target of radiation, normal tissues are inevitably irradiated. Radiation-induced injury usually encompasses two phases: an early phase characterized by tissue inflammation and a late phase characterized by tissue fibrosis. Depending on the irradiated site, organs develop radiation-induced injury, including the lung, liver, intestine and neurons. This section will discuss the role of CCL2 in radiation-induced injury.

#### Radiation-induced lung injury

Increased expression of CCL2 is related to radiation-induced toxicity in normal tissue [41]. In a mouse model of radiation‐induced pulmonary injury, both the selective CCL2 inhibitor bindarit (BIN) and knockout of the main CCL2 receptor CCR2 protected against normal lung tissue injury in mice. RT-induced vascular dysfunction and associated adverse effects were alleviated. RT-induced expression of CCL2 was abrogated by inhibiting CCL2 signalling. Lung fibrosis was delayed by long-term CCL2 signalling inhibition [41]. EMT has been reported to be involved in radiation-induced lung toxicity. Irradiated human pulmonary alveolar epithelial cells (AECs) (HPAEpiC cells) exhibited upregulated CCL2 and CCR4 expression. In vitro treatment with CCL2 induced EMT in HPAEpiC cells. This effect was weakened by the bioactive component of Radix Ophiopogonin B, CCR4 knockdown, or U0126 (a MEK/ERK inhibitor) [42]. However, not all radiation doses or modes promoted CCL2 secretion. Release of CCL2 from human umbilical vein endothelial cells (HUVECs) depended on the dose rate, and 4.1 mGy/h was optimal [43]. CCR2, the receptor for CCL2, is a marker of inflammatory monocytes. Mice deficient for CCR2 exhibited no pulmonary fibrosis after being irradiated and showed decreased numbers of infiltrating and interstitial macrophages [44]. Reactive oxygen species (ROS) have been implicated in radiation-induced pulmonary injury and fibrosis [45]. A study revealed that inducible nitric oxide synthase (iNOS) promoted the production of inflammatory cytokines such as CCL2 [46]. This finding suggests that radiation-induced injury was not due to a single factor but to the interaction between multiple factors.

#### Radiation-induced brain and liver injury

CCR2 has been shown to mediate cognitive impairments induced by irradiation. CCR2 knockout (-/-) mice received 10 Gy cranial irradiation. Compared with wild-type mice, CCR2 deficiency prevented hippocampus-dependent spatial learning and memory impairments induced by cranial irradiation. Moreover, CCR2 deficiency normalized the fraction of pyramidal neurons [47]. Irradiation to treat brain tumours could create a chronic neuroinflammatory state resulting in progressive cognitive decline. This effect was associated with the upregulation of CCL2, and inhibiting CCL2 signalling could attenuate this effect [48]. CCL2-deficient mice exhibited attenuated chronic microglial activation, which allowed for the recovery of neurogenesis. In radiation-induced liver damage, multiple chemokines, including CCL2, are released after irradiation. (Myo)fibroblasts in portal vessels may be one of the major sources of these chemokines [49].

Interestingly, not all radiation induces normal tissue injury. Compared with conventional RT (CRT), synchrotron microbeam radiation therapy (MRT) does not induce CCL2 expression in normal tissue [50]. This may explain why synchrotron MRT achieved equivalent tumour control as CRT but showed reduced normal tissue damage. This finding highlights the importance of radiation technology in radiation-induced injury.

### CCL2 as a therapeutic target

Based on these findings, CCL2 may be a therapeutic target or marker of normal tissue injury during irradiation. Numerous studies have investigated drugs that could prevent the release of radiation-induced inflammatory cytokines. Pravastatin is used to lower cholesterol, and it was found that pravastatin could limit the increase in blood CCL2 induced by radiation [51]. Fibroblast growth factor 2 peptide (FGF-P) is a peptide derived from the receptor-binding domain of fibroblast growth factor 2. In acute gastrointestinal syndrome (AGS) after radiation, FGF-P could reduce several proinflammatory cytokines, including CCL2 [52]. Inhibiting uPA and uPAR abrogated CCL2 expression in meningioma cell lines that were treated with radiation [53]. This outcome was accompanied by reduced ERK activation, nuclear translocation of Rel-A, and NF-κB-DNA binding activity. CCL2 is a marker of late rectal toxicity in prostate cancer patients undergoing RT. In patients with grade 2 late rectal toxicity, CCL2 levels were significantly increased [54]. Evaluating CCL2 levels at an earlier phase and targeting CCL2 may help prevent the development of rectal toxicity.

## CCL3

CCL3, also known as macrophage inflammatory protein-1α (MIP-1α), was first purified from the supernatant of endotoxin-induced murine macrophages [55]. Multiple cell types can produce CCL3, including immune cell subtypes, fibroblasts, and epithelial cells, as well as platelets, basophils, and eosinophils [56, 57]. CCL3 binds to CCR1, CCR4, and CCR5, exerting various effects on multiple immune cell subtypes. Due to the diverse functions of CCL3, it is involved in diverse biological processes.

### CCL3 is involved in the radiation-induced immune response

In a hepatocellular carcinoma model, mice treated with MIP-1α and irradiation exhibited significantly enhanced antitumour effects. Immunological markers, including CD8A and CD107A, were also increased [58]. In a phase I clinical trial, patients with breast cancer were treated with intratumoural H_2_O_2_ and RT. Blood marker analysis highlighted significant associations between MIP-1α and the tumour response [59]. This finding suggests that blood CCL3 may serve as a predictive marker for treatment response in specific cancer types.

### CCL3 and radiation-induced injury

CCL3 was reported to contribute to the development of radiation-induced injury. Both in vitro and in vivo experiments showed that CCL3 was increased by radiation. Mice deficient in CCL3 exhibited reduced inflammation, no significant septal thickening, and small collagen deposition foci in lung tissues after radiation. This effect was mediated by CCR1 but not CCR5. Both CCR1^−/−^ mice and the CCR1 inhibitor BX-471 ameliorated radiation-induced injury. CCR1 has been reported to promote Th2 cell recruitment. In CCR1^−/−^ mice, Th2 cytokines were not increased after irradiation. These results indicated that Th2 cytokines and cells are involved in the development of fibrosis [60]. Samples from radiated human conduit arteries exhibited sustained expression of inflammatory genes, including CCL3, and this radiation-induced CCL3 upregulation was due to NF-κB activation [61]. However, a study showed that in patients with prostate cancer who received three-dimensional conformal radiation therapy, MIP-1α levels were not sensitive to irradiation [62]. Based on these results, CCL3 could be a predictive biomarker for treatment response. This discrepancy in the effects of CCL3 may be attributed to the different responses in specific irradiated sites.

### CCL3 as a therapeutic target

Cryptotanshinone (CTS) is a major lipophilic extract from *Salvia miltiorrhiza* Bunge (Danshen). CTS can attenuate collagen deposition and pulmonary fibrosis in radiation-induced lung injury (RILI) rats by mitigating radiation-induced activation of CCL3 and its receptor CCR1 [63]. The reduced secretion of CCL3 alleviates the radiation-induced inflammatory microenvironment, subsequently attenuating the initiation of fibrosis. Since mitotic cells are highly sensitive to radiation, reducing the number of cells undergoing mitosis may be an ideal approach to protect normal tissues. MIP-1α induces a transient 50% reduction in the number of mitotic cells in small intestinal crypts [64]. BB-10010 is an analogue of MIP-1, and pretreatment with BB-10010 before irradiation significantly increased the number of surviving crypts (10%), which could prevent the side effects associated with RT [65]. This finding may support the use of BB-10010 in patients undergoing abdominal or pelvic treatments. ECI301 is a human MIP-1α variant. Daily administration of 2 µg of ECI301 for 3–5 consecutive days after local irradiation prolonged survival and eradicated tumour growth in CT26 and LLC models. The abscopal effect was observed, and this antitumour effect depended on CD8^+^ and CD4^+^ lymphocytes and NK1.1 cells [66].

## CCL5

CCL5 (also known as regulated on activation, normally T cell expressed and secreted (RANTES)) belongs to the C–C motif chemokine family. CCL5 binds to CCR5 with high affinity, but it can also bind to CCR1, CCR3 and CCR4 [67]. CCL5 can be found in cancer cells, CAFs, MSCs, MDSCs, TAMs, and anticancer tumour-infiltrating lymphocytes (TILs) [68]. The role of CCL5 in cancer is complicated; it has both protumour and antitumour properties. CCL5 is elevated in multiple tumours and indicates a poor prognosis [69]. However, studies have shown that CCL5 promotes antitumour immunity by recruiting antitumour T cells and DCs to the tumour microenvironment [67]. Thus, CCL5 is a double-edged sword in cancer.

### CCL5 promotes tumour progression

#### Tumour cell-derived CCL5

Macrophages are predominantly found in the tumour microenvironment. Although RT is effective at reducing primary tumours, it may enhance macrophage infiltration to tumour sites, accelerating tumour progression [70, 71]. In oesophageal squamous cell carcinoma, RT enhanced the expression levels of 12-LOX in tumour cells. Subsequently, CCL5 expression was upregulated through the AKT/NF-κB pathway. Elevated CCL5 can recruit macrophages to tumour tissues and promote their polarization to the immunosuppressive M2 subtype, thereby facilitating metastasis [72]. Similarly, radiation promotes macrophage migration in non-small-cell lung cancer models. CCL5 is responsible for this effect, as blocking CCL5 significantly reduces the number of migrated macrophages. Further analysis showed that CCL5 was upregulated by IL-6, and IL-6 inhibition impaired macrophage migration [35].

#### MSC-derived CCL5

MSCs play important roles in cancer metastasis and are involved in radiation-induced cancer lung metastasis. In the 4T1 breast cancer model, irradiated MSCs promote lung metastasis. The cGAS–STING signalling pathway is activated in irradiated MSCs. Knockdown of cGAS–STING signalling in MSCs abolished their prometastatic effect. Further analysis identified CCL5 as a downstream molecule. cGAS–STING pathway knockdown impaired CCL5 expression. Knockout of CCL5 in MSCs inhibited lung metastasis and CCR5^+^ macrophage infiltration. This finding highlights the role of the cGAS–STING-CCL5 pathway in metastasis [73].

Based on these results, CCL5 may indicate a worse prognosis in a specific context. A randomized phase 2 trial compared the efficacy of gemcitabine to capecitabine-based chemoradiotherapy in locally advanced pancreatic cancer. Baseline plasma samples were collected to identify circulating biomarkers. It was found that higher baseline circulating levels of CCL5 indicated worse overall survival [74].

### CCL5 promotes antitumour immunity

CD8^+^ T cell infiltration after RT is pivotal for tumour control. However, in a radiation-resistant mouse model, RT did not induce CD8^+^ T cell infiltration. CCL5 is involved in the recruitment of CD8^+^ T cells, which has been confirmed in solid tumours [75]. Studies have shown that CCL5 expression is much higher in parental tumour tissue than in radioresistant tumour tissue [76]. This finding highlights the role of CCL5 in CD8^+^ T cell infiltration.

Low CD8^+^ T cell infiltration and high programmed death ligand 1 (PD-L1) expression in pancreatic cancer predicts poor outcomes. Radiation combined with vaccination upregulates the expression of the chemokines CXCL10 and CCL5 in the tumour, along with increased CD8^+^ T cell infiltration. The use of anti-PD-L1 antibodies further enhanced the efficacy of the combination [77]. AZD6738 is an ATR inhibitor that has been investigated in early phase clinical trials. AZD6738 combined with RT significantly improved the efficacy of radiation. Increased infiltration of CD3^+^ and NK cells was observed in the tumour. In vitro and in vivo data demonstrated that ATRi plus RT promoted the production of CCL3, CCL5, and CXCL10 in tumour cells [78]. The released chemokines may contribute to the infiltration of antitumour immune cells. These results indicated that the presence of CCL5 promotes antitumour immunity and predicts a better response to treatment.

### CCL5 and radiation-induced injury

As mentioned previously, CCL2 contributes to radiation-induced EMT in HPAEpiC cells. CCL5 mediates this process, indicating that CCL5 may contribute to radiation-induced injury [42]. In human irradiated arteries, the inflammasome-associated chemokines CCL2 and CCL5 were elevated. In a mouse model of radiation-induced injury, the same effect was observed. Treatment with the IL-1 receptor antagonist anakinra significantly reduced CCL2 and CCL5 levels in mice. This finding indicated that IL-1 blockade could be a treatment for RT-induced vascular disease [79]. However, CCL5 not only contributes to radiation-induced injury but also protects normal tissue. Haematopoietic regeneration deficiency is a common side effect of RT. In mice with CCR5 deficiency in haematopoietic cells, haematopoietic regeneration was delayed. Further investigation showed that CCL5 was an endothelial cell-secreted haematopoietic growth factor that could bind to CCR5 on haematopoietic cells. CCL5 treatment accelerated haematopoietic regeneration. Mechanistically, CCL5 could promote haematopoietic cell cycling and survival [80]. These two experiments exhibited seemingly contrary results: CCL5 exerted a protective effect on haematopoietic regeneration, but in RT-induced cardiovascular disease, CCL5 promoted injury. This difference may be due to the different effects of CCL5 in different contexts. CCL5 binds to CCR5 on haematopoietic cells but not immune cells, thus promoting haematopoietic regeneration. In irradiated arteries, CCL5 may bind specific receptors on immune cells, thus promoting the development of inflammation.

## CCL8

CCL8 is also known as MCP-2. CCL8 can bind to CCR1, CCR2, CCR3, and CCR5 [81]. The main function of CCL8 is to recruit monocytes to inflammatory sites. In addition, CCL8 can attract eosinophils and Tregs [82]. In tumours, CCL8 can promote cancer cell proliferation [83] and migration [84, 85]. The role of CCL8 in radiation-related events is poorly understood. However, CCL8 may participate in lung metastasis. In a Lewis lung cancer model, thorax irradiation with 5 Gy X-ray dramatically increased the number of tumours in the lungs. The administration of nicaraven, a radioprotective agent, significantly reduced the number of tumours in the lungs. Moreover, radiosensitivity was not affected by nicaraven. Further investigation of the underlying mechanism revealed that nicaraven effectively inhibited CCL8 expression and macrophage recruitment [86]. Cranial irradiation can lead to cognitive impairments in patients with brain tumours. Radiation-induced inflammation may be the primary reason for these deficits. It has been reported that CCR2 is critical for macrophage trafficking in the brain. Gene expression analysis revealed radiation-induced gene expression of CCR2 ligands, including CCL8, in the hippocampus. CCR2 deficiency could reduce this induction and prevent hippocampus-dependent spatial learning and memory impairments [47]. This protective effect may be attributed to decreased infiltration of macrophages attracted by CCL8. In human irradiated arteries, CCL8 is dysregulated. This effect may be attributed to NF-κB activation [61]. However, the direct role of CCL8 in radiation-induced cardiovascular disease still needs further investigation.

## CCL11

CCL11 is a member of the eotaxin family and is also called eotaxin-1. CCL11 can recruit eosinophils by binding to the receptor CCR3 [7]. CCL11 is a ligand for CCR5 [87]. CCR3 was shown to be expressed in tumour cells, and activation of this receptor on cancer cells could increase proliferation and migration [88, 89]. Moreover, CCL11 could promote cancer cell apoptosis resistance [90] and angiogenesis [91]. A study showed that severe lung toxicity was associated with lower levels of eotaxin at 24 h after irradiation compared with that in patients who did not develop severe lung toxicity [92]. CCL11 could be a predictive marker of liver toxicity [93]. High-dose irradiation led to skin injury, and this effect was eosinophil-associated. Eosinophil-related Th2 cytokines, including CCL11, were upregulated in irradiated skin and HUVECs. Blocking IL-33 suppressed cytokine secretion in a coculture system of THP-1 cells and irradiated HUVECs, indicating that IL-33 may be a key molecule that regulates this process [94]. Similarly, in the context of radiation-induced brain tissue damage, inflammation-related genes, including CCL11, were strongly induced in endothelial cells [95]. In human dermal fibroblasts, the expression of eotaxin was upregulated by radiation, which may be an important step leading to eosinophilia in patients with radiation exposure [96]. Radiation-induced intestinal fibrosis (RIF) is a serious complication after abdominal RT. Excessive eosinophil accumulation is associated with RIF in both humans and mice. Irradiation increases extracellular adenosine triphosphate, which induces the expression of CCL11 by pericryptal α-SMA^+^ cells. Eosinophil-deficient mice showed marked amelioration of RIF. Blocking CCR3 by genetic deficiency or neutralizing antibodies suppressed eosinophil accumulation after irradiation in mice, suggesting a role for eosinophils in mice [97].

## Other chemokines

CCL7 is involved in the anti-inflammatory response and antitumour immunity [98, 99]. In fibrosis-sensitive C57BL/6 mouse model, total lung RNA was extracted at 26 weeks. CCL7 expression was upregulated, indicating a role in radiation-induced lung fibrosis [100]. CCL22 and CCL17 play pivotal roles in various type-2 T helper cell-dominant diseases. In a rat model of radiation pneumonitis/pulmonary fibrosis, CCL22 and CCL17 were upregulated in irradiated lung tissues. CCL22 and CCL17 were localized primarily to alveolar macrophages, as shown by immunohistochemistry. This finding indicated that macrophages are the primary producers of CCL22 and CCL17. Similarly, bronchoalveolar lavage fluid from patients with idiopathic pulmonary fibrosis exhibited elevated levels of CCL22. The corresponding receptor CCR4 was detected on alveolar lymphocytes and macrophages [101]. Thus, CCL22 and CCL17 are released by alveolar macrophages and may act on CCR4^+^ type-2 T helper cells and alveolar macrophages to promote pulmonary fibrosis. One of the biological functions of CCL20 is to attract immature DCs and lymphocytes to tissues. Injecting pcDNA3.1/MIP-3α into LLC subcutaneous tumours after radiation significantly delayed tumour growth. The accumulation of DCs was observed in tumour tissues. The local infiltrating lymphocytes after the treatment were predominantly CD8^+^ T cells [102]. Whole-body irradiation (WBI) depleted DCs in the epidermis and dermis. However, this effect was not due to DC apoptosis. Increased mRNA levels of CCL19/CCL21 and CCR7 in lymph nodes and skin were observed after radiation. CCR7 and its ligands CCL19/CCL21 mediate cDC migration to the draining lymph node. Thus, the radiation-induced depletion of DCs was due to DC migration to the lymph node. In vitro experiments showed that the number of DCs that migrated to CCL19-containing medium increased, indicating that chemokines regulated the migration of DCs [103]. However, a study showed that γ-ray irradiation could significantly inhibit the production of PEG2 and the expression of CCR7 induced by LPS. Eventually, the migration of DCs towards CCL19 is impaired in vitro and in vivo [104]. Low-dose RT (LD-RT) is known to exert an anti-inflammatory effect. In a coculture system of the EC line EA.hy.926 and polymorphonuclear neutrophils (PMNs), irradiation with a low dose between 0.5 and 1 Gy resulted in a significant reduction in CCL20 expression. Moreover, PMN/EA.hy.926 EC adhesion was significantly decreased [105]. In nasopharyngeal carcinoma, CCL22 was significantly increased in patient-derived xenograft tumours or cell lines upon radiation. In serum collected from patients who received RT, this effect was confirmed. Further experiments revealed that CCL22 could recruit CCR4^+^ CD8 T cells, which could enhance antitumour immunity [106]. Radiation could induce CCL27 production in the human keratinocyte cell line HaCaT. This effect depended on crosstalk between TNF-α and ROS. CCL27 production promotes skin immune and inflammatory reactions. However, the mechanism of CCL27 still needs investigation [107].

## Conclusion

CC chemokines mainly exert chemotactic effects, attracting monocytes, DCs, and T lymphocytes, which are involved in inflammatory reactions and antiviral effects. Radiation stimulates the production of these chemokines (Table 1). In this review, we mainly focused on the chemokines CCL2, CCL3, CCL5, CCL8, CCL11, CCL20, and CCL22. Multiple types of cells can produce CC chemokines, including tumour cells, endothelial cells, fibroblasts, and MSCs. The released chemokines bind to the corresponding receptors in an autocrine or paracrine manner. The most common effects of released chemokines were the recruitment of immune cells, including lymphocytes, macrophages, neutrophils and eosinophils, to irradiated sites, which could form an inflammatory environment. In normal tissues, this recruitment usually leads to acute and late radiation side effects. Radiation-induced chemokines can be detected in serum and thus could be used as predictive biomarkers. However, the time at which to measure serum levels of specific chemokines may differ in different cancer types. The optimal cut-off values of the predictive chemokines need to be critically evaluated. Several studies have identified relevant factors that inhibit specific chemokines to alleviate radiation-induced injury. This finding indicated that CC chemokines could be promising targets to inhibit the side effects of radiation. However, it should be noted that these strategies may influence the systematic inflammatory response, leading to serious side effects.

**Table 1:** Chemokines in radiation response

	Cell types	Producer (stimulator)	Recipient/signaling type (receptor)	Physiological effects	Reference
CCL2	Normal cell	Human pulmonary AEC cells	Autocrine (CCR4)	Lung fibrosis	[42]
	Normal cell	(Myo)fibroblasts	Neutrophiles	Liver damage	[49]
	Normal cell	Irradiated brain(not specific)	Microglial	CNS injury	[48]
	Cancer cell	A549, H157	Macrophage	Tumor promoting	[35]
	Cancer cell	Mouse pancreatic cancer cell lines	Inflammatory monocytes and macrophages	Radioresistance	[36]
	Cancer cell	HT1080, MPNST724, SK-LMS1,SW684	Th1/Tc1 T cells	Tumor control	[38]
	Cancer cell	TC1 cells	Monocytes and CCR2(+) regulatory T cells	Radioresistance	[39]
	Cancer cell	MC38, LLC cells	CCR2(+) MDSC	Radioresistance	[37]
	Cancer cell	CNE2, HONE1, SUNE2 cells	Cancer cell	Radioresistance, metastasis and epithelial-mesenchymal transition	[40]
CCL3	Normal cell	Irradiated lung tissue (not specific)	CCR1(+) CD4 and CD8 T cells, and macrophages	Radiation-induced lung injury	[60]
	Normal cell	Human conduit arteries	Not specific	Cardiovascular disease after irradiation	[61]
CCL5	Normal cell	Human pulmonary AEC cells	Autocrine (CCR4)	Pulmonary fibrosis	[42]
	Normal cell	Endothelial cells	CCR5(+) hematopoietic cells	Hematopoietic regeneration	[80]
	Cancer cell	Eca109, Kyse150 cells	Macrophages	Cellular Metastasis	[72]
	Cancer cell	Mesenchymal stem cells	CCR5(+) macrophages	Lung Metastasis	[73]
	Cancer cell	A549, H157 cells	Macrophage	Tumor promoting	[35]
	Cancer cell	B16 cells	CD8+ T cell	Tumor control	[76]
CCL8	Normal cell	Irradiated lung tissues (not specific)	Macrophage	Lung metastasis	[86]
	Normal cell	Irradiated hippocampal (not specific)	Not specific	Cognitive impairments	[47]
CCL11	Normal cell	Pericryptal alpha-SMA(+) cells	CCR3(+) Eosinophil	Radiation-induced intestinal fibrosis	[97]
	Normal cell	Irradiated skin	Eosinophil	Radiation-induced skin injury	[94]
	Normal cell	Dermal fibroblasts	Eosinophil	Eosinophilia	[96]
	Normal cell	Endothelial cells	Not specific	Radiation-induced brain tissue damage	[95]
CCL20	Cancer cell	LLC cells	DC, lymphocytes	Tumor control	[102]
CCL22	Normal cell	Human NPC cell lines, patient-derived tumor xenograft tumors	CCR4(+) CD8 T cell	Tumor control	[106]
	Normal cell	Irradiated lung tissues(not specific)	Alveolar lymphocytes and alveolar macrophages	Radiation pneumonitis/pulmonary fibrosis	[101]

In tumour tissues, the infiltration of these immune cells, especially macrophages, can promote metastasis or radioresistance. However, in some cases, anticancer effector cells are recruited to facilitate tumour control. Some chemokines, such as CCL2 and CCL5, exert both protumour and antitumour effects. Thus, it is difficult to target these chemokines as a treatment strategy. The protective effects of specific chemokines have been reported, which could exert induce antitumour immunity. To maximize the antitumour effects of chemokines, radiation doses, fractions or techniques should be taken into consideration. The patterns of chemokine release may differ in response to different doses or fractions. Both preclinical experiments and clinical trials are needed to confirm the optimal doses to boost antitumoural chemokines.

Although the role of chemokines in radiation is complex, investigation of the responses of the chemokines to RT may lead to the development of novel treatments.

## Data Availability

Not applicable.

## Abbreviations

cGAS-STING: Cyclic GMP-AMP synthase stimulator of interferon genes
CAF: Cancer-associated fibroblasts
CRT: Conventional radiotherapy
DC: Dendritic cells
DNA: Deoxyribonucleic acid
DSB: Double-strand breaks
EMT: Epithelial-mesenchymal transition
FGF-P: Fibroblast growth factor 2
HUVECs: Human umbilical vein ndothelial cells
IFN: Interferon
iNOS: Nitric oxide synthase
MCP-1: Monocyte chemoattractant protein 1
MDSCs: Myeloid-derived suppressor cells
MIP-1: Macrophage inflammatory protein
MIP-1α: Macrophage inflammatory protein-1α
MSC: Mesenchymal stem cells
MRT: Synchrotron microbeam radiation therapy
NF-B: Nuclear factor kappa-light-chain-enhancer of activated B cells
NPC: Nasopharyngeal carcinoma
PDAC: Pancreatic ductal adenocarcinoma
PD-L1: Programmed death ligand 1
TAMs: Tumor-associated macrophages
TAN: Tumor-associated neutrophils
TILs: Tumor-infiltrating lymphocytes
Treg: Regulatory T lymphocytes
RT: Radiotherapy
TNF: Tumor necrosis factor-alpha
RANTES: Regulated on activation, normally T cell expressed and secreted
RIF: Radiation-induced intestinal fibrosis
ROS: Reactive oxygen species
